# The Role of Plant Growth Promoting Rhizosphere Microbiome as Alternative Biofertilizer in Boosting *Solanum melongena* L. Adaptation to Salinity Stress

**DOI:** 10.3390/plants11050659

**Published:** 2022-02-28

**Authors:** Souhair Mokabel, Zakia Olama, Safaa Ali, Rehab El-Dakak

**Affiliations:** 1 Department of Botany and Microbiology, Faculty of Science, Alexandria University, Alexandria 21511, Egypt; souhair.mokabel@alexu.edu.eg (S.M.); zakia.olama@alexu.edu.eg (Z.O.); 2 Nucleic Acid Research Department, Genetic Engineering and Biotechnology Research Institute, City of Scientific Research and Technology Applications (SRTA-City), Alexandria 21934, Egypt; sali@srtacity.sci.eg

**Keywords:** biofertilizer, biopesticide, eggplant, gene expression, physiological traits, polyamines, salinity, stress, *Solanum melongena*

## Abstract

Recent ecological perturbations are presumed to be minimized by the application of biofertilizers as a safe alternative to chemical fertilizers. The current study aims to use bioinoculum (I) as an alternative biofertilizer and to alleviate salinity stress in the cultivar *Solanum melongena* L. Baldi. The salinity drench was 200 mM NaCl (S), which was used with different treatments (0; I; S; S + I) in pots prefilled with clay and sand (1:2). Results showed that salinity stress inhibited both plant fresh and dry weights, water content, and photosynthetic pigments. The content of root spermine (Spm), spermidine (Spd), and puterscine (Put) decreased. However, addition of the bioinoculum to salt-treated plants increased pigment content (80.35, 39.25, and 82.44% for chl a, chl b, and carotenoids, respectively). Similarly, K+, K+/Na+, Ca2+, P, and N contents were significantly enhanced. Increases were recorded for Spm + Spd and Put in root and shoot (8.4-F, 1.6-F and 2.04-F, 2.13-F, respectively). RAPD PCR showed gene expression upregulation of photosystem II D2 protein, glutathione reductase, glutathione-S-transferase, protease I, and protease II. The current work recommends application of the selected bioinoculum as a green biofertilizer and biopesticide. Additionally, the studied eggplant cultivar can be regarded as a source of salt tolerance genes in agricultural fields.

## 1. Introduction

Chemical fertilizers have been widely used to achieve maximum crop productivity in conventional agricultural systems. Nevertheless, when chemical fertilizers exceed the threshold level, they accelerate soil acidification, pollute groundwater, and harm the environment overall [1]. It is recommended to apply biofertilizers as eco-friendly alternatives as they play a pivotal role in phosphate solubilization, nitrogen fixation, production of ammonia, enzymes, siderophores, and secretion of variable phytohormones. Furthermore, they exhibit biocontrol activity against a wide variety of phytopathogenic agents [2]. Bhattacharyya and Jha [3] observed that inoculation of bacteria and fungi into the soil or seedling roots may colonize either the rhizosphere or the inner sections of the plants, thus enhancing plant growth and development.

The application of microbes can be used to enhance systemic tolerance in plants against biotic as well as environmental stress. *Bacillus subtilis* inhibits pathogens causing diseases either directly or indirectly through a biocontrol mechanism [4]. Some *Pseudomonas* species stimulate plant growth through the production of water-soluble vitamins like niacin [5]. Besides, specific mycorrhizal fungi like *Trichoderma harzianum* that act as plant symbionts can be widely used for their ability to induce plant tolerance to biotic and abiotic stresses such as salinity and drought [6]. Moreover, inoculation of *Aspergillus terreus* enhances NaCl tolerance in *Pennisetum glaucum* by mitigating the physicochemical attributes of the host plant [7]. Interestingly, the plant growth promoting ability of *Pinicillium critinum* initiates habitat revegetation and conservation. Also, its production of GA_5_ from gibberellins opens new insights in research and investigation [8]. 

Eggplant (*Solanum melongena*) is the fifth most economically important crop within the Solanaceae family and is considered by the Food and Agriculture Organization of the United Nations (FAO) as one of the 35 foods with the most considerable relevance for world food security [9]. In this respect, Egypt is ranked third out of the top five producing countries of eggplant [10]. Because eggplant has a relatively long growth period, it is more exposed than other vegetable crops to a broad range of plant diseases, pests, nematodes, and weeds [11]. 

Excess salts in soil pose a serious threat to agricultural production and environmental health [12]. Soil salinity is a widespread problem that extends over one billion hectares spread across many countries. If left abandoned, this condition could develop into a socioeconomic and human health problem in the long run [13].

Eggplant is a sensitive species to salinity and is not well adapted to saline soils [14]. Thus, one of the objectives of this study was to select the best combination of plant growth promoting rhizobcteria (PGPR) and rhizo fungi (RF) for eggplant growth in saline soil and investigate the alleviative role of combining the selected bioinoculum on salinity stress in *Solanum melongena* L. Baldi cultivar. Another objective was to elucidate the physiological and molecular systemic resistance exhibited by eggplant to withstand salt stress. Finally, a question was posed to establish whether the selected bioinoculum applied is recommended (or not) in agricultural fields as a biofertilizer/biopesticide alternative to chemical fertilizers.

## 2. Results

### 2.1. Molecular Identification and Phylogeny of Bacterial Isolates in the Selected Bioinoculum

**Figure 1:**
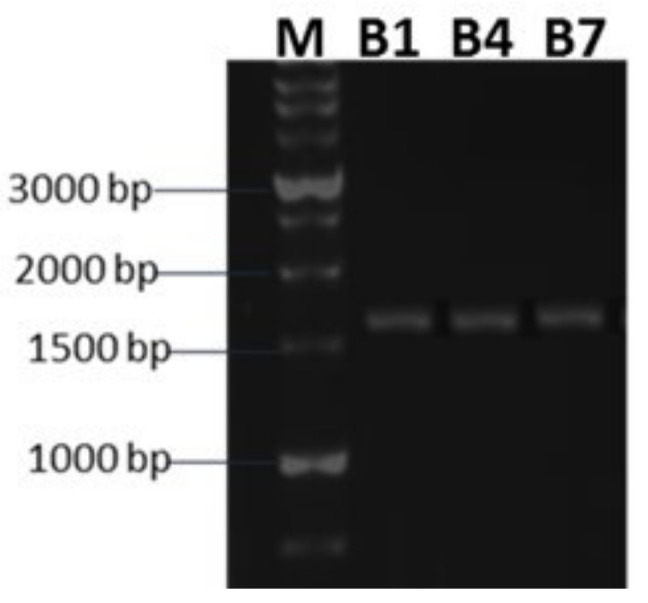
Agarose gel electrophoresis of the amplified PCR fragment for 16 r DNA gene. M: 1 kb DNA marker, isolates with code no. (B1, B4 and B7) (MW~1550 bp).

**Figure 2:**
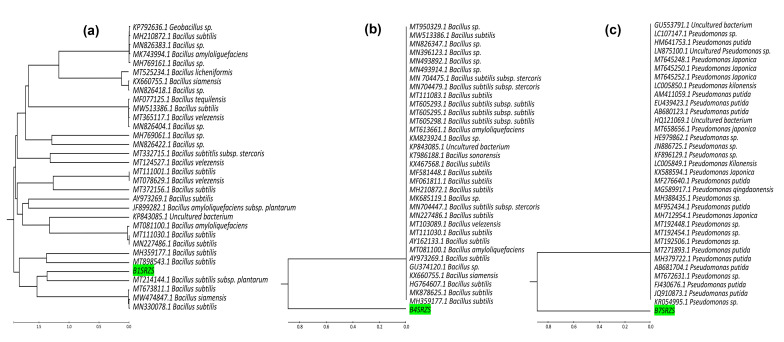
Phylogenetic tree of isolate coded B1SRZS, B4SRZS, and B7SRZS ((**a**–**c**), respectively) achieved, displaying the location amid the selected bacteria based on 16Sr RNA sequence assessments.

Identification of the most promising isolates, B1, B4, and B7, was carried out using 16S rDNA (Figure 1). Agarose gel (1%) was used to examine the PCR results, where the 1500 bp amplified fragment’s sequence was easily determined. The sequences of various types of strains from the gene bank were a popular tool for identifying and classifying prokaryotes. Thus, when the gene sequences (16 s) of the selected bacterial isolates were compared to those of the gene bank, the isolate code B1 (B1SRZS) was found to be 99.71 percent identical to *Bacillus subtilis* with accession no. MT214144.1 (Figure 2a), the isolate code B4 (B4SRZS) was found to be 99 percent identical to *Bacillus subtilis* with accession no. MH359177.1 (Figure 2b), and the isolate code B7SRZS was found to be 99 percent identical to *Pseudomonas sp.* with accession number KR054995.1 (Figure 2c). Supplementary Tables S3–S5 show the percentages of similarity and accession numbers obtained after comparing the sequences of the tested strains to the sequences submitted to Gene Bank.

### 2.2. Solanum melongena L. Salt Tolerance Indices Percentage as Affected by Salt Stress and/or Inoculum Treatments

*Solanum melongena* L. seeds were germinated and grown in sandy clay soil for 66 days and treated with the selected PGPR and RF alone and in combination with 200 mM NaCl. At the end of the experimental period, the role of selected inoculum of PGPR and RF was assessed in morphometric traits including plant height (PH), root length (RL), shoot length (SL), R/S ratio, individual and total root and shoot; fresh and dry weights (RFW; RDW; SFW; SDW; TFW; TDW), and leaf area (LA), in addition to individual and total root and shoot water content (RWC; SWC; TWC). Salinity stress significantly decreased leaf area, root fresh and dry weights, in addition to root water content by (59.14, 95.87, 96.30, and 95.66%, respectively) compared to control (Supplementary Data—Tables S1 and S2). Inoculating the soil of salt-stressed eggplants showed a significant increase in leaf area, total fresh, dry weight, and water content (by 1.95-F, 3.53-F, 3.97-F, and 3.42-F, respectively) compared to salt-stressed plants (Supplementary Data—Tables S1 and S2). Salt tolerance indices percentage (STI%) revealed that the selected inoculum significantly increased salinity tolerance of eggplant morphometric traits except for PH, RL, SL, R/S ratio, and LA (Table 1). The significant increase in STI% was recorded for RFW, SFW, TFW, RDW, SDW, TDW, RWC, SWC, and TWC (17.67-F, 2.98-F, 4.90-F, 19.88-F, 3.79-F, 7.37-F, 16.82-F, 2.82-F, and 4.4-F, respectively). 

### 2.3. Solanum melongena L. Leaves Photosynthetic Pigments and Chlorophyll Fluorescence as Affected by Salt Stress and/or Inoculum Treatments

The effect of the selected microbiome on photosynthetic pigments revealed that under salinity stress all pigments were significantly decreased compared to control (Table 2). Nevertheless, inoculating *S, melongena* L. plants after exposure to salinity stress resulted in a significant increase in chl a, chl b, chl a + b, chl a/b, and carotenoid content by (80.35, 39.25, 69.20, 29.37, and 82.44%, respectively) compared to salt stressed plants. 

Examination of the maximum quantum efficiency and photochemical efficiency of PSII using chlorophyll fluorescence expressed by F_v_/F_m_ and F_v_/F_0_ unexpectedly showed insignificant change among all treatments tested.

### 2.4. Element Homeostasis in Solanum melongena L. as Affected by Salt Stress and/or Inoculum Treatments

**Figure 3:**
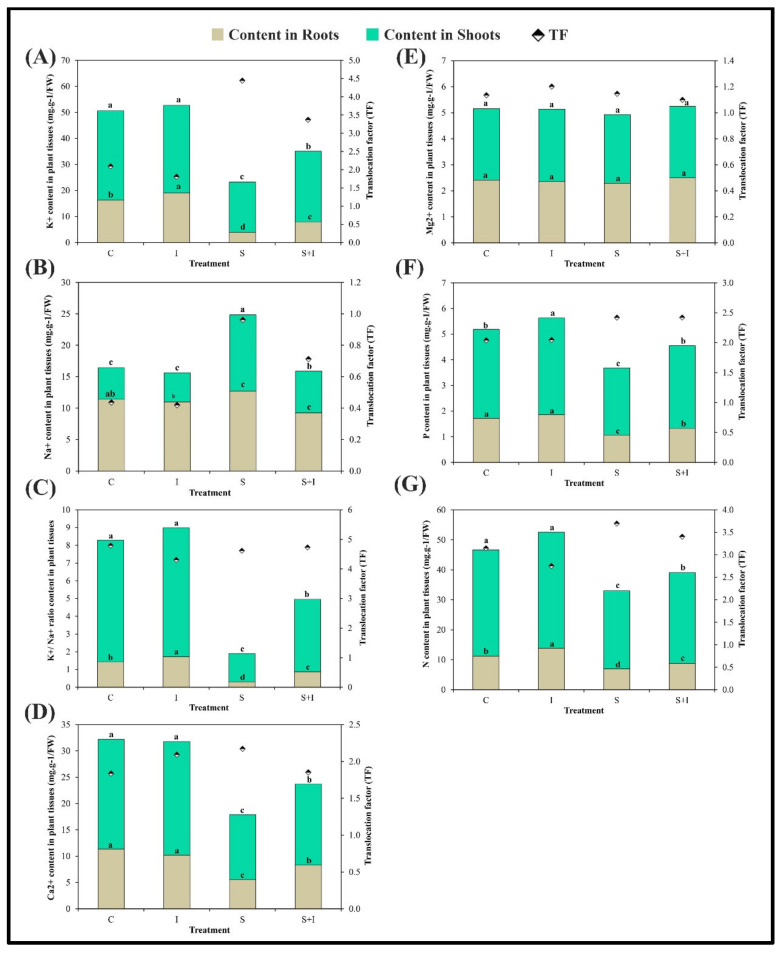
Effect of 0 (C), selected bioinoculum (I), 200 mM NaCl (S), and their interactions on elements content: (**A**) = potassium (K+), (**B**) = sodium (Na+), (**C**) = K+/Na+ ratio, (**D**) = calcium (Ca2+). (**E**) = magnesium (Mg2+), (**F**) = phosphorous (P), and (**G**) = nitrogen (N) and translocation factor (TF) (mineral content in shoots/mineral content in roots) in the roots and shoots of *S. melongena* L. plants 66 days after sowing. Values are means ± SD based on triplicate independent determinations, and different letters means a significant difference as evaluated by Duncan’s multiple comparison test (*p* < 0.05).

To detect the elemental homeostasis in *S. melongena* plants, the element contents (K^+^, Na^+^, K^+^/Na^+^, Mg^2+^, Ca^2+^, P, and N) of shoots and roots were determined (Figure 3). Under salinity stress, a significant decrease was observed for all ions except for Na^+^ compared to control. In contrast, no significant effect of Mg^2+^ was recorded for *S. melongena* root or shoot. In the case of the double interaction S + I, there was a significant increase for K^+^, K^+^/Na^+^, Ca^2+^, P, and N ions (2, 2.8, 5.6, 1.2, 1.3-F in root and 1.4, 2.6, 1.3, 1.2, 1.16-F in shoot, respectively). Whereas, Na^+^ showed an opposite trend to the other measured ions compared with salt-stressed plants.

Interestingly, ions such as K^+^, K^+^/Na^+^, Ca^2+^, P, and N were found to be transported actively from roots to shoots (Translocation factor (TF) = 3.4, 4.7, 1.9, 2.4, and 3.4, respectively) at S+I treatment, whereas Na^+^ attained relatively lower values (TF = 0.71 and 0.9, respectively). The relative high translocation factor for measured ions in inoculated *S. melongena* after salinity stress suggests the presence of a transport system somehow for these ions from root to shoot as a defense mechanism towards salt stress.

### 2.5. Hormonal Status in Salt-Stressed Solanum melongena L. as Affected by Salt Stress and/or Inoculum Treatments

Quantification of free polyamines in *S. melongena* L. plants showed that salinity-stress significantly decreased Spmand Spd. levels in roots (by 100 and 68, respectively), while it increased Spm and Spd levels in shoots by 26% and 113%, respectively with respect to control (Table 3). Inoculation with selected PGPR and RF caused a marked increase in levels of Spm + Spd and Put in the root as well as the shoot (8.4-F, 1.6-F and 2.04-F, 2.13-F, respectively) compared to salt-stressed plants. It is also notable that root Put (2.37 mg·g^−1^FW) was greater than Spm + Spd (0.84 mg·g^−1^FW) in bioinoculated salt stressed eggplants compared to S, whereas shoot Put (0.17 mg·g^−1^FW) attained lower values than Spm + Spd (1.14 mg·g^−1^FW).

To investigate the mechanisms related to osmotic adjustment, it is necessary to incorporate measurement of proline content in roots and shoots of *S. melongena* under normal and stressed conditions. The results presented in Table 3 showed that there was an increase in proline content under salinity stress by 57% and 30% in root and shoot, respectively, compared to control. Application of the selected bioinoculum to salt-stressed eggplants resulted in a significant increase in proline content of roots and shoots by 18.50% and 23.31%, respectively, compared to salt-stressed pots.

### 2.6. Selected Inoculum Modulates Some Genes Related to Defense System of Salt-Stressed Solanum melongena L. Roots and Shoots

The measurements of parameters at the molecular levels are essential to understand the defense mechanism of inoculated eggplant after exposure to salinity stress.

**Figure 4:**
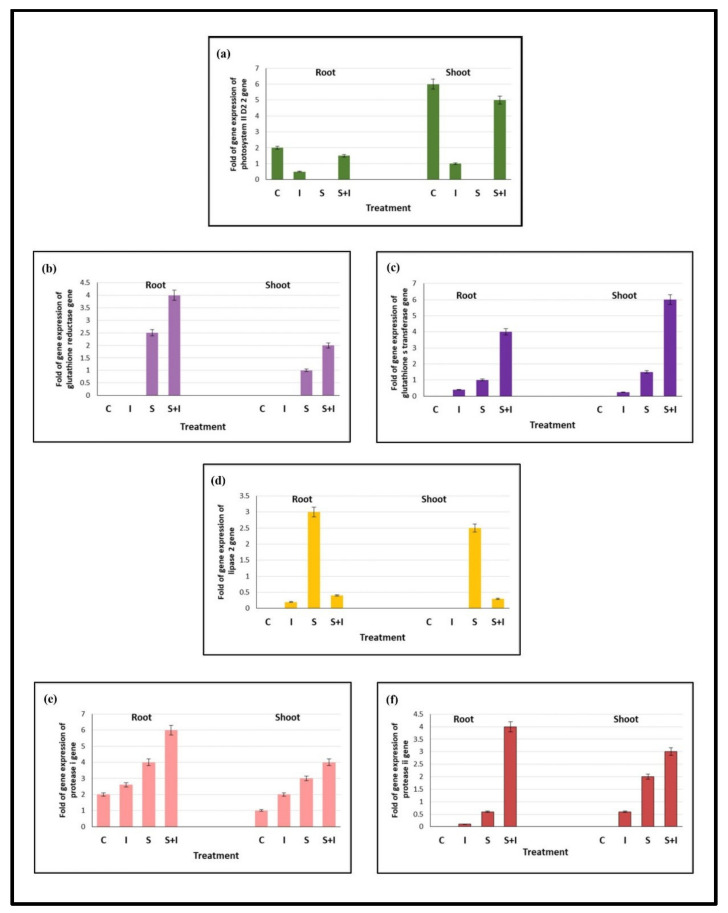
Effect of 0 (C), selected bioinoculum (I), 200 mM NaCl (S), and their interactions on the gene expression of PSII D2 (**a**), glutathione reductase (GR) (**b**), glutathione S transferase (GST) (**c**), lipase (**d**), protease I (**e**), and protease II (**f**) in the roots and shoots of *S. melongena* L. plants 66 days after sowing. Values are means ± SE based on triplicate independent determinations.

The fold of gene expression for five genes related to different physiological aspects— photosynthesis, glutathione, lipids, and proteins—is represented in Figure 4. These genes are related to variable physiological aspects: photosynthesis, glutathione, lipids, and proteins. 

The gene of psbD related to D2 protein participates in the D1/D2 heterodimer of PSII complex where it binds with cofactors to facilitate e-transfer for ATP formation (Figure 4a). The gene expression of shoot psbD was upregulated about 5-fold after inoculation of salt-stressed eggplants. 

Glutathione reductase (GR) plays an important role in the ascorbate–glutathione cycle under abiotic stress. The gene expression of GR in the current study was increased by about 4-fold and 2-fold for root and shoot, respectively at S + I compared to salt stressed pots (Figure 4b).

Glutathione S-transferase (GST) minimizes reactive molecules with the accumulation of glutathione (GSH), thus protecting the cell from oxidative damage. The gene expression of GST recorded upregulation by about 4-fold and 6-fold for roots and shoots, respectively, with respect to salt-stressed pots (Figure 4c).

Lipases cause alterations in membrane lipid composition; this response induces the formation of toxic lipid intermediates that cause membrane damage or cell death. Unlike all the tested genes, the gene expression of lipase was downregulated at S + I in roots and shoots by 86.7% and 88%, respectively, compared to salt-stressed eggplants (Figure 4d). 

In plants, PGPR can produce cell-wall-degrading enzymes (e.g., proteases) to suppress pathogen growth. In the present study, the gene expression of Prot. I and II was upregulated in inoculated salt-stressed plants with respect to stressed pots. It is to be noted that Prot. I showed higher gene expression upregulation than Prot. II for root and shoot (Figure 4e,f).

## 3. Discussion

*Solanum melongena* L. is an important economic species that exhibits a sensitive response to moderate salinity levels [15]. 

In the present study, application of 200 mM NaCl caused a significant decrease to all morphometric growth traits except RL and R/S ratio. Several studies have shown an inhibition in growth parameters and biomass production due to the increase in salinity level [15,16]. *Triticum vulgare* exhibited reduction in plant height and total shoot dry weight under 200 mM NaCl-stress [17]. Perica et al. [18] reported that the primary response to salinity stress is expressed by reduction of root and shoot development. In the current work, a relatively insignificant increase in root length accompanied by a significant decrease in shoot length emphasized that shoot growth is more sensitive to salt stress than root. Therefore, an increase in R/S ratio is a direct consequence of plants exposure to salinity-stress [19].

The applied selected inoculum to salt-stressed plants in the present study caused a significant increase in morphometric traits such as: root and shoot water contents together with fresh and dry weights. As Ha-Tran et al. [20] elucidated, the beneficial impacts of crop plant bacterization with PGPR include inducing plant growth and development and stimulating tolerance to salt stress through different mechanisms. Also, PGPR contributes to salinity stress alleviation in plants by advancing water absorption ability and enhancing essential nutrients uptake [21]. Abd Allah et al. [22] found that the *B. subtilis* (BERA71)-inoculated salt-stressed *Cicer arietinum* plants yielded higher plant biomass with reduced ROS levels compared to the non-inoculated seedlings. Concomitantly, Khosravi et al. [23] reported that the use of *Pseudomonas fluorescens* bacterium enhanced shoot dry matter and uptake of different nutrients in *Lactuca sativa* L.

In the current work, reduction of photosynthetic pigments is another consequence of salinity stress that led to photosynthetic disruption. This finding agreed with the results of Taibai et al. [24] on *Phaseolus vulgaris* L., and on *Vigna subterranean* L. [25]. Lower levels of photosynthetic pigment under salt stress were attributed to either slow synthesis or fast degradation of pigments [26]. Nevertheless, the results of all photosynthetic pigments were significantly increased when the selected bioinoculum was applied to salt-stressed plants. Photosynthetic pigments of *Gossypium hirsutum* were significantly reduced under salinity conditions, however, utilization of salt tolerant bacteria including *Bacillus subtilus* and *Bacillus sp.* while producing IAA restored cotton systemic resistance to salt-stress [27]. Analogously, the protective effects of salt tolerant PGPR on photosynthetic pigment were also recorded in *Phaseolus vulgaris* L. and *Arachis hypogaea* L. [28]. Azarmi-Atajan et al. [29] reported that inoculating *Lactuca sativa* L. with strains of *Pseudomonas sp.* significantly increased chlorophyll and carotenoids content under different salinity levels, enriching soil fertility through the production of hormones, proteins and prolines [30]. This may elucidate the role of the selected bioinoculum in the enhancement of chla, chl b, and carotenoids under salt-stress conditions, as well as suggest that this microbiome can be applied as an effective green biofertilizer under salt-stress conditions. 

Insignificant changes in PSII activity expressed by F_v_/F_m_ and F_v_/F_0_ in all treatments suggest that the activity of PSII isn’t affected by salinity stress. This is supported by four eggplant regenerates that showed an evident stability in tolerating salinity by preserving quite stable F_v_/F_m_ Chl fluorescence [31]. Similar findings have been attained for salt-tolerant tomato [32] and wheat [33]. The insignificant increase in Chl fluorescence was accompanied by a significant increase in photosynthetic pigments after microbiome inoculation of salt-stressed plants. Inoculation with *Azotobacter chroococcum* and application of Zn^2+^ to Glycine max causes enhancement of chlorophyll (a, b, and a + b), carotenoid contents, and F_v_/F_m_ ratio [34]. 

The current work’s elemental status revealed that the utilized bioinoculum significantly restricted Na^+^ uptake in the roots of salt stressed plants, with TF = 0.71 compared to 0.96 under salt stress. In addition, the decreased Na^+^ uptake was possibly due to a reduced passive (apoplasmic) flow of Na^+^ into the stele as a result of a greater proportion of the root zones being covered with soil sheaths in inoculated treatments [35,36]. However, K^+^ ions and K^+^/Na^+^ ratios were increased more in shoots than roots. The favored translocation of K^+^ over Na^+^ from roots to shoots and the greater K^+^/Na^+^ ratio in the shoots of inoculated eggplant indicated that selective uptake of K^+^ had occurred, which appears to be one of the processes involved in the tolerance of eggplant to salinity stress. Similar results were detected by Ha-Tran et al. [20] on NaCl-exposed eggplants inoculated with *Bacillus brevis*. Moreover, *Bacillus subtilis* (BERA 71) stimulates the acquisition of K^+^, Ca^2+^, Mg^2+^, and N in *Cicer arietinum*, while inhibiting Na^+^ uptake [22]. 

The significant increase of the selected bioinoculum to K^+^, K^+^/Na^+^ and Ca^2+^ revealed that this association collaborated to expel Na^+^ ions. This might be concluded from the perspective mechanism illustrated by El-Dakak et al. [37] involving salt overly sensitive signaling pathway (SOS). Thus, the proposed regulatory mechanism to control salinity stress in eggplant involves the mitigating effect of PGPR and RF through decreasing Na^+^ and increasing K^+^ as both share a common pump. Thus, K^+^/Na^+^ ratio as well as Ca^2+^ content increased where both have an effective role in expelling Na^+^ through SOS pathways. An increase in Ca^2+^ content gets perceived by SOS3, which starts the regulation mechanism of ion homeostasis. Then, SOS3 binds to the activated SOS2 [38]. The activated SOS3–SOS2 protein complex phosphorylates the SOS1 plasma membrane Na^+^/H^+^ antiporter to efficiently pump Na^+^ out of the cell [39].

The slight insignificant increase in Mg^2+^ ions for shoots of inoculated salt-stressed eggplants was accompanied by a significant increase in photosynthetic pigments. This may be explained by the fact that in the current study, Mg^2+^ concentration was recorded at 2.75 mg g^−1^ DW which might not hamper photosynthesis. In addition, it was reported by Hauer-Jákli and Tränkner [40] that Mg^2+^ concentrations of less than 1.5 mg g^−1^ DW didn’t induce negative effects on the photosynthetic capacity of *Triticum aestivum* and *Helianthus annus*. In another study, low leaves Mg^2+^ supply (0.3 mg g^−1^DW) did not affect vegetative biomass formation in wheat [41]. 

Phosphorous and nitrogen were also significantly enhanced in bioinoculated salt- stressed eggplants compared with salinity-stressed conditions. This was suggested to be due to the attractive approach of P-solubilizing activity of *Bacillus subtilis*, *Pseudomonas sp.*, and *Aspergillus terreus*. *Bacillus subtilis* solubilizes soil P, enhances nitrogen fixation, and produces siderophores that suppress the growth of pathogens [42]. The *Pseudomonas stutzeri* strain A1501 fixes nitrogen under microaerobic conditions in the free-living state where certain gene products in this species are involved in the regulation of the nitrogen fixation process. Interestingly, *A. terreus* strains were able to solubilize zinc-P under very saline conditions (up to 10% NaCl) [43]. 

*Solanum melongena* as glycophyte restricts toxic ions intake by roots and constrains their transport to the shoot. In the current study, under salinity stress Na^+^ ions were largely translocated to the shoot; this was revealed from the relatively higher TF compared to that of untreated plants, while application of selected bioinoculum to salt-stressed eggplants relatively decreased the TF of Na^+^ ions with respect to saline pots. The *Solanum trovum* resistance mechanism to salinity was moderately based on the active transport of toxic ions to the leaves and, probably, a better capacity to store them in the vacuoles [44,45].

Polyamines are documented to enhance plants stress tolerance, however, PGPR secretion of polyamines is largely unknown [46]. Application of the selected bioinoculum to salt-stressed eggplants in the current work showed a significant increase of both root and shoot tested Spm and Spd compared to salt stressed plants. The shoot attained relatively higher PAs than the root, suggesting production of hormonal signals from the root that communicates information to the shoot. The higher level of root Put compared to Spm + Spd revealed its substantial alleviative role in defending against salt-stress in eggplant roots. Because of the polycationic nature of polyamines, they participate with antioxidant activity and scavenging free radicals in plant salt tolerance strategies to abiotic stresses [47]. Concomitantly, Zhang et al. [48] emphasized Put effect on enhancing salt tolerance by reducing the oxidative damage in *Glycine max* L. roots. The exogenous Put might be closely related to salt stress tolerance in plants, which inhibits Na^+^ uptake and stimulates K^+^ inward, thus increasing the K^+^/Na^+^ ratio in cucumber [49]. Current data showed that the increase in endogenous root Put exerted the same effect on Na^+^, K^+^, and K^+^/Na^+^ ratio. 

Nevertheless, the shoot Spm + Spd of the studied eggplant exceeded Put by about 7-fold suggesting their substitutive role in shoot salt tolerance. Zapata et al. [50] recorded an increased ratio of (Spm + Spd)/Put under salinity that has been correlated with the defensive role against salt-stress. Similarly, El-Shintinawy [51] also referred that salinity significantly boosted the increase of Spm and Spd accompanied with an inhibition in Put content in *Triticum aestivum* L. cultivars. Xie et al. [52] reported that *Bacillus subtlilis* OKB105 synthesized spermidine and is the pivotal compound related to plant growth promotion. Additionally, Spermidine from *Bacillus megaterium* BOFC15 increased cellular polyamine accumulation in *Arabidopsis*, thereby activating PA-mediated signaling pathways contributing to the osmotic stress tolerance of plants [46].

Accumulation of proline is a common feedback to salt stress in crop plants. In the current study, under salinity stress, proline content recorded a significant increase in roots and shoots compared to control. Proline content was increased in two genotypes (Barcelone and Threa) of *S. melongena* L. under salinity stress [53]. Similarly, leaf proline levels increased significantly in *S. melongena* and *S. insanum* in response to different salt stress treatments, moreover it is suggested that *S. insanum* is more tolerant to salinity stress due to its capacity to accumulate proline and to a lesser extent Na^+^ and Cl^−^ [44]. 

Application of the bioinoculum to salt stressed eggplants resulted in higher proline content for roots and shoots, which was consistent with the substantial enhancement of proline after inoculation of *Oryza sativa* with nine salt tolerant bacterial isolates including *Bacillus sp.* [54]. This clarifies the mitigating role of proline in the defense system of eggplants through sweeping ROS, adjusting ion homeostasis, protecting osmotically, and stabilizing subcellular structures. Interestingly, it seems to be a cooperative role between proline and polyamines to alleviate salinity stress in roots and shoots of *S. melongena* L. as both attained increasing contents at S + I compared to salt-stressed pots, at least in the presented dataset under the prevailing experimental conditions. 

Molecular characterization of the gene expression using RAPD PCR reveals some of the physiological defensive mechanisms. The expression of psbD gene encoding the D2 subunit of the PSII reaction center (PSII D2 protein) is particularly interesting, because D2 represents the starting point for the assembly of PSII as a whole [55]. In the current work, the upregulation of the shoot psbD gene expression by about 5-fold was achieved after treatment of salt-stressed eggplants with the selected bioinoculum. This refers to the efficiency of binding to cofactors and the effectiveness of electron transfer through D2 protein that constitutes part of the D1-D2 heterodimer of PSII; consequently, it signifies the efficiency of PSII in ATP formation. The amount of D2 available directly determines the levels of the other component subunits of PSII via feedback control mechanisms according to the CES model (control by epistasis of synthesis) for the temporal sequence of PSII assembly [56]. As well, mycorrhizal colonization of salt stressed *Robinia pseudoacacia* upregulated the expression of three chloroplast genes, including RppsbD in leaves [57].

The activity of GR is reported to play a pivotal role in determining stress tolerance in plants under different abiotic stresses [58]. The upregulation of GR gene expression in eggplant roots and shoots confirmed that, as a consequence of PGPR and RF inoculation, eggplant acquired protection against challenging salt stress. These results agree with the study of Gururani et al. [59], who documented the enhanced mRNA expression of different ROS pathway genes after exposure to salt, drought, and heavy metal stress in PGPR inoculated *Solanum tuberosum* plants. Also, inoculating *Abelmoschus esculentus* L. plants with PGPR, including *Bacillus megaterium* and *Enterobacter sp.* resulted in the upregulation of ROS pathway genes (APX, CAT, DHAR, and GR) under salinity stress [60]. Moreover, in a study by Brenes et al. [44], results of semiquantitative RT-PCR revealed that expression levels of different ROS pathway genes encoding APX, CAT, GR, and DHAR were increased in the ACC deaminase-containing PGPR treated salinized plants compared to the untreated controls. 

Various environmental stimuli enhanced the expression of GST, including biotic stresses such as fungal elicitors and pathogen attack [61], and abiotic stresses such as salt [62] and drought [63]. The gene expression of GST in the currently studied salt-treated eggplants inoculated with PGPR and RF was upregulated 4-fold in salt-treated pots. Trehalose metabolite application derived from PGPR improved glutathione-S-transferase (GST), CAT, APX, and DHAR activities, and stimulated the glyoxalase network [64]. Analysis of Arabidopsis cell-suspension culture suggested early stress-induced changes in the expression of genes with the involvement of GST in oxidative stress protection [65]. Similarly, GmGSTU4-expressing transgenic tobacco plants provided tolerance to salt stress and the herbicide alachlor [66]. 

Different types of abiotic stress, including salinity, induce lipid remodeling through specific lipases that form toxic lipid intermediates, causing membrane damage or cell death [67,68]. Nevertheless, in the current study, downregulation of lipase gene expression for salt-stressed eggplants treated with bioinoculum confirmed the mitigating role of PGPR and RF of salt stress through the integrity of lipid constituents of the cell membrane. Singh and Jha [69] discovered that treating salt-stressed wheat plants with the *Serratia marcescens* bacterium efficiently scavenged ROS, resulting in lower levels of lipid peroxidation and membrane injury. It was reported in a study by Cook et al. [70] that lipases play a pivotal role in oxylipins synthesis, thus lipases specific to lipids of thylakoids confer in environmental stress response. Therefore, it has been suggested to focus on the role of lipases under different environmental stresses to better understand the physiological regulatory mechanisms involved.

Control of pathogenic diseases can be generated by PGPR extracellular secretion of proteases that hydrolyze other microbial cell walls, and trigger induced systemic resistance against pathogenic infection in plants [71]. Thus, in the current work, the exposure of salt-stressed eggplants to the selected bioinoculum caused the production of two proteolytic enzymes (prot. I and prot. II) with higher upregulation of prot. I than prot. II in both roots and shoots. This suggests that the biocontrol of PGPR and RF as biopesticides against infectious pathogens could enhance the systemic resistance of eggplants. Aminisarteshnizi [72] emphasized that the growth-promoting bacterium *Pseudomonas fluorescens* produces a lytic enzyme protease that suppresses the root-knot pathogen nematode *Meloidogyne incognita* in eggplant crops. In addition to inhibiting penetration and consequent root-knot infection in *Vigna radiata* [73], *Bacillus subtilis* strain and *Pseudomonas aeruginosa* caused more than 50% mortality of juveniles of the root-knot nematode *Meloidogyne javanica*. Also, an isolated strain of *Bacillus subtilis* was recorded to induce systemic resistance against powdery mildew on barley [74]. In a study by Behera et al. [75], the two fungal isolates *Trichoderma longibrachiatum* and *Penicillium rubidurum* were able to produce a proteolytic zone of the alkaline protease. Moreover, many fungal species of the genera *Aspergillus*, *Trichoderma*, *Rhizopus*, *Mucor*, and *Penicillium* have been documented for protease production [76].

**Figure 5:**
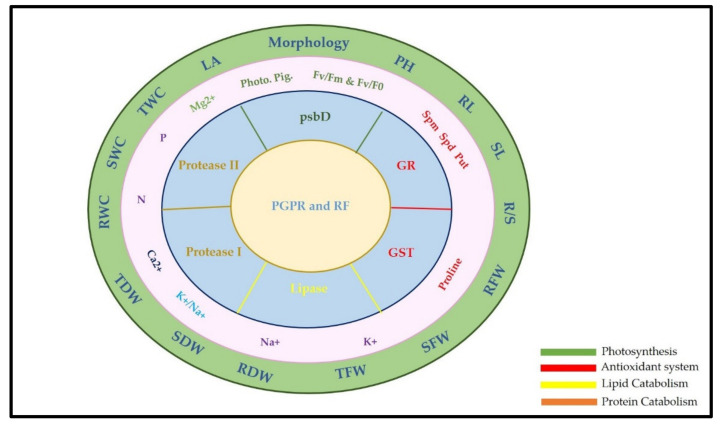
Proposed modulation of systemic resistance in salt-stressed *Solanum melongena* L. shoots and roots due to inoculation with selected PGPR and RF. Colored box refers to physiological aspects; psbD related to photosynthesis in green; GR and GST related to antioxidant system in red; lipase related to lipid catabolism in yellow; protease I and protease II related to protein catabolism in brown.

The PGPR and RF selected in the current work suggest a modulatory impact on the systemic resistance of *Solanum melongena* L. Baldi cultivar to challenging salt stress, which strongly recommends their application as an effective green biofertilizer and biopesticide. Systemic resistance begins with plant genes until morphometric traits (Figure 5). Resistance originates from gene expression related to four of the most treasured physiological aspects in plants, photosynthesis, the antioxidant system, and the catabolism of lipids and proteins. Thus, the collaboration of these genes was reflected in the biochemical constituents in roots and shoots, and sometimes leaves. It seems that psbD enhances photosynthetic pigments and the efficiency of PSII (F_v_/F_m_ and F_v_/F_0_) with no significant change among all treatments. Osmoprotection and antioxidant systems to ROS formed under salinity stress are scavenged and controlled by GR and GST, which might have a role in increasing proline and polyamines content. Elemental homeostasis contributes to either restricting or expelling Na^+^ ions via the SOS signaling pathway. Catabolism of lipids contributes to preserving the compartment cell membrane while defending against pathogenic diseases and is achieved by lytic proteases. Finally, this was expressed in the morphology and morphometric traits of *Solanum melongena* L. plants.

## 4. Materials and Methods

### 4.1. Experimental Materials and Growth Conditions

A pure variety of eggplant seeds (*Solanum melongena* L. Baladi) cultivar was obtained from Nubaseed Company. Viable seeds of eggplant were surface sterilized with 0.1% (*v*/*v*) HgCl_2_ solution for 5 min and then rinsed three times with distilled water. Seeds were soaked in distilled water for 5 days to break dormancy until they radically began to rise a few mm in length. Early germinated seeds (EGS) were translocated to a styrofoam seedling tray. The soil mixture used in all experiments was composed of (clay: sand; 1:2). The styrofoam seedling tray was watered twice a week with distilled water till the first foliage leaf appeared (14 days after sowing “DAS”). 

### 4.2. Isolation and Identification of Plant Growth Promoting Rhizobacteria and Rhizofungi

Strains of PGPR as well as RF were isolated from the rhizospheric soil of *S.melongena* L. Baladi cultivar grown in saline soil. In experiments conducted as part of the current study, two strains of PGPR (*Bacillus subtilis* B1ZSRS and *Pseudomonas sp.* B2ZSRS) and three strains of rhizofungi (RF) (*Trichoderma harzianum*, *Aspergillus terrus*, and *Penicillium citrinum*) were used as test inoculum. 

#### 4.2.1. Differentiation between Bacterial Isolates

RAPD PCR was developed in recent years to describe and indicate the phylogeny of different organisms. The chosen bacterial isolates were subjected to RAPD PCR utilizing A and C short primers; their sequences are A: AGGAGGACACTATGAGTG and C: TACGGYACCTTGTTACGACTT. The cyclic reaction, composed of 4 min at 95 °C and then 40 cycles of 40 s at 94 °C, 50 s at 30 °C, and 50 s at 72 °C, followed by a supplementary 10 min at 72 °C [77]. Primer C more effectively differentiated selected bacterial isolates than primer A. For all isolates tested, the size of the amplified PCR fragment was estimated; the primers will bind somewhere in the structure, but it is unknown exactly where. Three bacterial isolates, B1, B4, and B7 were subjected to further study and identification.

#### 4.2.2. Molecular Identification and Phylogeny of Bacterial Isolates

A Thermo Fisher’s kit was used to extract genomic DNA. The universal primers were used to amplify the 16S rRNA region (F: AGAGTTTGATCMTGGCTCAG and R: TACGGYACCTTGTTACGACTT) [78]. The reaction was carried out with the aid of a DNA template. The MEGA 7 software tool was used to perform several alignments based on the most closely related sequences and similarity values. The MEGA 7 program was used to rebuild a phylogenetic tree.

#### 4.2.3. Identification of Fungal Strains

The three fungal strains used in the applied bioinoculum were identified at the mycological center in the faculty of science at Assuit University, Egypt. The fungal cultures were grown in sterile Petri plates containing autoclaved Czapek’s yeast extract agar (CYA) followed by incubation for 7 days at 28°C [79]. The medium contained (g/L): sucrose, 30; Na_2_NO_3_, 2; K_2_HPO_4_, 1; KCl, 0.5; MgSO4.7H_2_O, 0.5; FeSO_4_, 0.01; ZnSO_4_, 0.01; CuSO_4_, 0.005; yeast extract 5; chloramphenicol, 0.25; and agar, 15 (final pH 7.3). Identification of the growing fungi was based on colony characteristics (growth rate, color, texture, and reverse pigmentation) as well as microscopic features (shape of conidiophores, conidiogenous cells, and conidial dimensions). Fungal hyphae and conidia were stained with lactophenol cotton blue for better visualization. An Axiostar trinocular microscope, made by Zeiss in Germany, was used for the examination. The main references consulted for identification included [80,81,82].

### 4.3. Characterization of the Selected Plant Growth Promoting Bacteria and Rhizofungi 

Table 4 gives an insight into different bacterial and fungal species constituting the selected bioinoculum applied to the *S. melongena* Baldi cultivar exposed to salinity stress in the present study.

### 4.4. Experimental Design

The experiments of the current study were carried out as factorial experiments based on Randomized Complete Block Design, with three replications in the Botanical Garden at the Faculty of Science, Alexandria University, Egypt. A sublethal concentration of 200 mM NaCl was determined after preliminary salinity screening of 30–300 mM NaCl on *Solanum melongena* L.

The tested inoculum (2 PGPR X 3 RF) was selected based on prior permutations and combinations using Minitab (version 12) for different PGPR and RF strains that have been isolated from *S. melongena* as previously mentioned. The tested isolates were purified, identified, and numbered, then preserved on 50% glycerol stock. Biochemical analysis of isolated PGPR and RF was carried out before identification.

Selection of the tested inoculum applied in the current work on *S. melongena* under salinity stress was based on the best morphological parameters measured and the leaves proline content from a preliminary experiment (Supplementary Table S1). 

The experiments were carried out under controlled conditions (photon flux density (PFD) of 450 µmol m^−2^·s^−1^, 14/10 h light/dark cycle, temperature of 30 ± 5 °C and relative air humidity of about 85%). After sowing, the field capacity of the culture pots was estimated at 450 mL of demineralized water. Experiments were carried out using a homogenous dried soil mixture in plastic culture pots (12 cm diameter and 30 cm height) with 1 kg soil capacity. In each pot, two selected seedlings from the styrofoam seedling tray of *S. melongena* were planted (14 DAS). Pots were regularly irrigated to field capacity three times/week during the whole experiment. Planted pots were left for 9 days to acclimatize to pot conditions, and on day 9, pots were supplied with 1/4 N Hoagland solution. The first half of the pots received distilled water (30 DAS), while the second half received 200 mM NaCl until wilting symptoms appeared. All pots were exposed to optimized closed growth chambers.

There were 40 pots for each experimental unit (replicate), with 2 plants/pot. The pots were divided into four sets (47 DAS) with 20 plants/set; one irrigated with distilled water I; the second one inoculated with the selected inoculum and irrigated with distilled water (I); the third set was irrigated with 200 mM NaCl (S); and the fourth set was irrigated with 200 mM NaCl and inoculated with selected inoculum (S + I). Eggplant samples were harvested 66 days after sowing, washed with running tap water, followed by demineralized water, then blotted gently using layers of tissue paper.

### 4.5. Measurement of Growth Traits

Different growth traits were determined in shoots and roots at the end of the salt treatments: plant height (PH), root length (RL), shoot length (SL), R/S ratio, and fresh weight of root and shoot systems were immediately determined. Dry weight samples were dried at 80 °C till a constant dry weight was reached. The salt-tolerance index (STI %) for different treatments was calculated as the percentage of the ratio of the value for the NaCl-treated plant/value for the control [98]. STI was calculated for all measured growth traits.

The Leaf Area Meter, a Model LI 3000 Portable Area Meter assembled with a conveyor belt, was used to calculate leaf area. Individual areas were measured to the nearest square centimeter.

### 4.6. Extraction and Estimation of Chlorophyll and Carotenoid Contents

According to the method of Lichtenthaler et al. [99], the content of chlorophyll and carotenoids were determined. *Solanum melongena* leaves fresh weight (100 mg FW) was blotted dry on tissue paper and placed in 5 mL of di-methyleformamide, left to stand in the dark over night for complete extraction. Absorbance was recorded at 646.8 nm and 663.2 nm for the chlorophyll assay and 453 nm for the carotenoids assay (in the supernatant) by a UV-Visible spectrophotometer (JENWAY, 6305, Staffordshire, ST15 OSA, UK).

### 4.7. Measurement of Chlorophyll Fluorescence

Chlorophyll fluorescence measurements were monitored in fully expanded young leaves. Measurements of Chl fluorescence were performed with the OS-30P pulse modulated chlorophyll fluorimeter (Opti-sciences, Hudson, NY, USA). Fluorescence was excited by illuminating leaves with a wear, red pulsed measuring light intensity (<0.1 μmol m^−2^s^−1^) with a peak wavelength of 650 nm. Prior to measurement of fluorescence, plants were kept in darkness at 22 ± 2 °C for at least 40 min to allow dark adaptation to ensure that the primary quinine acceptor (QA) was maximally oxidized. The basal non-variable chlorophyll fluorescence level with open PII reaction centers (F_o_) and the maximal fluorescence intensity indicator (F_m_) level with closed PSII were determined at room temperature on intact leaves of 10 replicate plants from all treatments. The F_o_ (as initial fluorescence level) was measured by a weak red measuring beam, followed by a saturation light pulse to measure the maximum F_m_ level. The variable fluorescence (F_v_) was calculated as the difference between F_m_ and F_o_. The maximum quantum yield of PSII (F_v_/F_m_) was also calculated [100]. 

### 4.8. Elements Analysis

Sample preparation, metal analysis, and quality control were carried out according to the standard method of Kimbrough and Wakakuwa [101]. Oven-dried and homogenously milled, 200 mg of plant samples were mixed with 3 mL of concentrated HNO_3_ in a beaker and covered with a ribbed watch glass. Then, the mixture was heated on a hot plate at 90–95 °C and left to evaporate to a low volume. After cooling, the previous step was repeated with additional portions (3 mL) of HNO_3_ until the digested solution either turned into a lighter color or reached a stable color, and the digestate was then refluxed with a small portion of HCl (3 mL) for complete digestion. Finally, the sample was filtered through filter paper (Whatman 42, diameter 110 mm). Then, the beaker walls and watch glass were washed with deionized water, and the filter paper was rinsed with diluted HNO3 (10%). The final volume was adjusted to 25 mL with deionized water. To determine different metal contents, the solutions were subjected to Inductively Coupled Plasma-Optical Emission Spectroscopy (ICP-OES; Agilent 5100 VDV, Santa Clara, CA, USA). The content of Na^+^, K^+^, Ca^2+^, and Mg^2+^ was computed as mg·g^−1^. The flow rates of plasma, auxiliary, and nebulizer of ICP-OES were kept at 12, 1, and 0.7 mL·min^−1^, respectively. The sample uptake and stabilization time were 10 s for each sample.

### 4.9. Polyamines Detection

Detection of free polyamines (spermine, spermidine, and puterscine) in *S. melongena* L. shoots and roots were carried out according to the method described by Gong and Liu [102] using HPLC Amens 2/001.

### 4.10. Differential-Display Reverse Transcription-PCR (DDRT-PCR) and Semi Quantitative Gene Expression

#### 4.10.1. RNA Extraction

The frozen plant tissue was transferred to an appropriately sized RNase-free tube. The Easy-spin™ Total RNA Extraction Kit was used to extract total RNA from plant tissue. A High-Capacity cDNA Reverse Transcription Kit was used for the generation of the first cDNA strand. 

#### 4.10.2. RAPD-PCR

Twelve short primers specific for photosystem II D2, glutathione reductase (GR, EC 1.6.4.2), glutathione-S-transferase, lipase, protease I, and protease II genes designed using sequences in the gene bank were subjected to PCR for cDNA amplification (Table 5). The resulting patterns were analyzed using a statistical method to determine the molecular weight of different bands that appeared on the agarose gel. PCR reaction was performed for 4 min at 95 °C followed by 40 cycles each of: 40 s at 94 °C, 50 s at 30 °C, and 50 s at 72 °C, followed by a supplementary 10 min at 72 °C. After amplification by PCR, the products were checked on 2% agarose gel electrophoresis. Bands which give molecular weight specific to each gene were compared to all treatments (root and shoot) and semi-quantitative analysis was carried out using software of gel analyzer (Syngene Geneflash Gel Documentation). A confirmation test was done for the specific PCR product under molecular weight related to the gene after purification using a gel extraction purification kit using the pair of primers that related to the gene under test. A PCR reaction was performed for 4 min at 95 °C followed by 40 cycles each of: 40 s at 94 °C, 50 s at 55 °C, and 50 s at 72 °C, followed by a supplementary 10 min at 72 °C using pairs of primers for specific gene(s) (Table 5). After converting nucleotide sequences to amino acid sequences, sequence data were analyzed and compared with data from a gene bank using different bioinformatics programs (Tcoffee and BioEdit). 

### 4.11. Statistical Analysis

A two-way analysis of variance (ANOVA) approach was used to analyze the obtained data. Duncan’s multiple comparison range tests using SPSS software [103] were carried out to identify statistically significant differences among the treatments at *p* < 0.05. Data were presented as means ± standard deviation (*n* = 3), with different alphabetical letters revealing significant differences between treatments. Student *t*-test was used for normally distributed quantitative variables to compare between two studied groups. “*” means statistical significance at *p* ≤ 0.05. The translocation factor (TF) of elements in plants was calculated by dividing the element content in shoots by the element content in roots [104]. Statistical analysis software followed the methods of Sokal and Rohlf [105].

## 5. Conclusions

Most of the studies emphasize the role of plant growth promoting rhizobacteria against biotic and abiotic stresses. Nevertheless, the current study revealed the synergistic effects between rhizofungi and PGPR in salt-stress mitigation, which surprisingly enhance pathogenic disease resistance concomitant with inherent systemic resistance in *Solanum melongena* L. The ameliorative role of polyamines was depicted in enhancing resistance in eggplant roots and shoots; as putrescene was activated to defend root salinity stress, it was substituted by spermine and spermidine in shoot salt resistance. Further studies are suggested to elucidate the PAs signaling communication for plant-bioinoculum crosstalk. Molecular examination at the gene level untangled the systemic resistance of *S. melongena* to salt-stress with different physiological aspects, which necessitates more investigation to unravel the physiological mechanisms and pathways involved. Thus, the *Solanum melongena* L. Baldi cultivar could be regarded as a source of “salt-tolerance” genes for the genetic improvement of this trait in other eggplants. In particular, improvement of almost all eggplant measured parameters was achieved through PGPR and RF inoculation after 17 days of exposure to 200 mM NaCl (until wilting). Future application of concomitant inoculum with salt stress predicts much more improvement, as this mimics the probability of further saline water irrigation in agricultural fields. The experiments conducted to test the efficiency of rhizobacteria and rhizofungi revealed that the applied bioinoculum is attractive as well as an economic approach for sustainable agriculture. In the new climate change scenario, there is a need to lower the use of chemical fertilizers. Thus, the abrupt shift towards the environmentally safe, more productive use of natural biofertilizers to reduce pest attacks is a demand of time. Therefore, it is recommended to be addressed in open field conditions to save eggplant as one of the most important economic crops worldwide. Furthermore, it is not just a biofertilizer to counteract salt-noxious effects, but it also acts as a biopesticide due to fungal resistance to pathogenic diseases. The agricultural world is now eager for such types of associations to be applied to further economically important crop plants.

## Figures and Tables

**Table 1:** *Solanum melongena* L. salt tolerance indices percentage as affected by 200 mM NaCl (S) and/or inoculum (I) treatments.

STI% of Parameters	S	S + I	T	*p*
PH	76.89 ± 11.62	82.41 ± 11.65	0.581	0.592
RL	118.61 ± 28.20	112.53 ± 24.45	0.282	0.792
SL	60.35 ± 12.71	70.93 ± 17.23	0.856	0.440
R/S	204.65 ± 69.85	167.30 ± 59.26	0.706	0.519
RFW	4.12 * ± 0.55	72.79 ± 39.92	2.979 *	0.041 *
SFW	16.97 * ± 5.20	50.56 ± 6.48	7.002 *	0.002 *
TFW	11.03 * ± 1.54	54.07 ± 11.72	6.310 *	0.022 *
RDW	3.68 ± 0.33	73.14 ± 36.09	3.333 *	0.029 *
SDW	17.35 * ± 2.93	65.79 ± 15.08	5.459 *	0.005 *
TDW	9.12 * ± 0.23	67.21 ± 20.81	4.834 *	0.040 *
RWC	4.35 * ± 0.71	73.15 ± 42.58	2.798 *	0.049 *
SWC	16.90 * ± 5.69	47.71 ± 5.13	6.964 *	0.002 *
TWC	11.64 * ± 1.98	51.18 ± 9.91	6.774 *	0.002 *
LA	41.24 ± 5.95	92.68 ± 35.89	2.449	0.128

Note: STI % = salt tolerance index percentage; PH = plant height; RL = root length; SL = shoot length; R/S = root/shoot ratio; RFW = root fresh weight; SFW = shoot fresh weight; TFW = total fresh weight; RDW = root dry weight; SDW = shoot dry weight; TDW = total dry weight; RWC = root water content; SWC = shoot water content; TWC = total water content; and LA = leaf area. Parameters measured 66 days after sowing. Values are means ± SD based on triplicate independent determinations, * means statistically significant difference at *p* ≤ 0.05, t: Student *t*-test.

**Table 2:** *Solanum melongena* L. leaves photosynthetic pigments as affected by 200 mM NaCl (S) and/or inoculum (I) treatments.

Parameters	Treatments			
	C	I	S	S + I
Chl a (μg g−1 FW)	10.11 ^a^ ± 0.78	7.38 ^b^ ± 0.61	5.75 ^b^ ± 0.77	10.37 ^a^ ± 2.39
Chl b (μg g−1 FW)	3.30 ^a^ ± 0.38	1.99 ^c^ ± 0.11	2.14 ^bc^ ± 0.09	2.98 ^ab^ ± 0.82
Chl a + b (μg g−1 FW)	13.41 ^a^ ± 0.74	9.36 ^b^ ± 0.71	7.89 ^b^ ± 0.85	13.35 ^a^ ± 3.20
Chl a/b	3.06 ^b^ ± 0.23	3.71 ^a^ ± 0.02	2.69 ^c^ ± 0.08	3.48 ^a^ ± 0.05
Carot. (μg g−1 FW)	2.36 ^a^ ± 0.24	1.78 ^b^ ± 0.11	1.31 ^b^ ± 0.12	2.39 ^a^ ± 0.49
F_v_/F_m_	0.79 ^a^ ± 0.01	0.79 ^a^ ± 0.03	0.75 ^a^ ± 0.02	0.76 ^a^ ± 0.02
F_v_/F_0_	3.84 ^a^ ± 0.27	3.76 ^a^ ± 0.60	2.98 ^a^ ± 0.36	3.38 ^a^ ± 0.31

Note: C = control; Chl a = chlorophyll a; Chl b = chlorophyll b; Chl a + b = chlorophyll a and b; Chl a/b = chlorophyll a/b; Carot. = carotenoids; Fv/Fm = variable to maximum fluorescence; and Fv/F0 = variable to ground fluorescence. Parameters measured 66 days after sowing. Values are means ± SD based on triplicate independent determinations, and different letters means significant difference as evaluated by Duncan’s multiple comparison test (*p* < 0.05).

**Table 3:** Hormonal Status in *Solanum melongena* L. roots and shoots as affected by Salt Stress and/or inoculum treatments.

Parameters	Treatment							
	Roots				Shoots			
	C	I	S	S + I	C	I	S	S + I
Spm (mg g^−1^FW)	0.58 ^b^ ± 0.01	0.0 ^c^ ± 0.0	0.0 ^c^ ± 0.0	0.66 ^a^ ± 0.02	0.31 ^d^ ± 0.02	0.80 ^b^ ± 0.04	0.39 ^c^ ± 0.02	0.93 ^a^ ± 0.02
Spd (mg g^−1^FW)	0.31 ^a^ ± 0.02	0.0 ^d^ ± 0.0	0.10 ^c^ ± 0.01	0.18 ^b^ ± 0.01	0.08 ^c^ ± 0.02	0.10 ^c^ ± 0.01	0.17 ^b^ ± 0.01	0.21 ^a^ ± 0.02
Put (mg g^−1^FW)	3.37 ^a^ ± 0.07	0.18 ^d^ ± 0.01	1.50 ^c^ ± 0.03	2.37 ^b^ ± 0.02	0.08 ^b^ ± 0.01	0.03 ^c^ ± 0.01	0.08 ^b^ ± 0.02	0.17 ^a^ ± 0.02
Prol (µg g^−1^ FW)	10.35 ^c^ ± 0.28	7.73 ^d^ ± 0.67	16.22 ^b^ ± 0.30	19.22 ^a^ ± 1.22	18.71 ^c^ ± 1.43	8.87 ^d^ ± 0.26	24.37 ^b^ ± 0.29	30.05 ^a^ ± 0.54

Note: C = control; Spm = spermine; Spd = spermidine; Put = putersciene; and Prol = proline. Parameters measured 66 days after sowing. Values are means ± SD based on triplicate independent determinations, and different letters means significant difference as evaluated by Duncan’s multiple comparison test (*p* << 0.001).

**Table 4:** Microbial load (bioinoculum I) characterization applied on *Solanum melongena* L. after exposure to 200 mM NaCl salinity stress.

Organism	Type of Organism	Location of the Organism	Function of the Organism	Reference
*Bacillus subtilis*	Gram + ve non-pathogenic Bacterium	Soil/Colonizing plant roots	* Salt tolerant bacterium * Protect cellular membranes integrity * Increase nitrate reductase and glutamine synthetase activities * Supply IAA to the cultures * Reduce ethylene generation under salt stress through ACC deaminase secretion, thus increase nutrient uptake and growth. * Decrease oxidative and osmotic induced stress * Manifested plant growth improvement, slowing down statolite starch hydrolysis under salinity	[4,83,84,85,86]
*Pseudomonas sp.*	Gram -ve Bacterium	Saprophytic/ parasite on plant surfaces	* Salt tolerant bacteria * Promote plant growth by suppressing pathogenic micro-organisms * Synthesize growth-stimulating plant hormones * Promote increased plant disease resistance	[87,88,89]
*Trichoderma harizanum*	Free-living saprophytic fungi	In most types of soils/mutualistic endophytic with plant species	* Salt tolerant fungi * Significantly suppress the growth of plant pathogenic microorganisms * Regulate the rate of plant growth * Well known for biological control mechanism * Produce secondary metabolites in agroecosystems	[90,91,92]
*Aspergillus terrus*	Saprophytic filamentous fungi	Part of the soil microbiota/can be found in many types of soils/frequently found as endophytic	* May live at pH 3 and 30% salinity * Used with PGPR, induces positive effects on plant growth and development * Induce systemic resistance and reduce plant stress * Attain phosphorous-solubilizing activity * Strong biocontrol activity	[93,94,95]
*Penicillium citrinum*	Mesophilic Fungus	Soil is their natural habitat	* Isolates tolerate salt concentration above 10% * Plant growth promoting ability * Contain ACC deaminase activity which sustains plant growth and development under stress conditions * Produces mycotoxin citrinin, cellulase, endoglucanase, as well as xylulase. * Produces Gibberellins	[8,96,97]

**Table 5:** Primers used for the cDNA amplification to detect photosystem II D2 (psbD), glutathione reductase (GR), glutathione-S-transferase (GST), lipase (Lip.), protease I (Prot. I), and protease II (Prot. II).

Primer Sequence	Length (bp)	Gene
F: AGGCTGTGGACCGACATCTA	266	psbD
R: GCTCATGAACACGTCCCTCT		
F: CACATCCTGATCGCCACCG	200	GR
R: TCCTTCCTGAAGCACAGGTC		
F: GAAGATCCCCGTGCTGATCC,	390	GST
R: AAGTTGGGGAACTTCTCGCT		
F: GCACATCCTGAGGGTGAACA	369	Lip.
R: AGCTCGTAGTCCTCCCTGTC		
F: AGGCTGTGGACCGACATCTA,	489	Prot. I
R: GCTCATGAACACGTCCCTCT		
F: CGACACCATGCAGTACGTGA,	386	Prot. II
R: TGGCGTAGTTGGCGTACATC		

## Data Availability

The data presented in this study are available on request from the corresponding author.

## Supplementary Materials

The following supporting information can be downloaded at https://www.mdpi.com/article/10.3390/plants11050659/s1, Table S1: Effect of 0 (C), selected bioinoculum (I), 200 mM NaCl (S) and their interactions on plant height (PH), root length (RL), shoot length (SL), root/shoot ratio (R/S), root fresh weight (RFW), shoot fresh weight (SFW), total fresh weight (TFW) of *Solanum melongena* L. plant 66 days after sowing; Table S2: Effect of 0 (C), selected bioinoculum (I), 200 mM NaCl (S) and their interactions on root dry weight (RDW), shoot dry weight (SDW), total dry weight (TDW), root water content (RWC), shoot water content (SWC), total water content (TWC), and leaf area(LA) of *Solanum melongena* L. plant 66 days after sowing; Table S3: Similarity percentages and accession numbers obtained after comparing the sequence of the tested strain (B1SRZS) to the submitted sequences in Gene Bank; Table S4: Similarity percentages and accession numbers obtained after comparing the sequence of the tested strain (B4SRZS) to the submitted sequences in Gene Bank; Table S5: Similarity percentages and accession numbers obtained after comparing the sequence of the tested strain (B7SRZS) to the submitted sequences in Gene Bank.
