# Supplementary material for: The Role of Plant Growth Promoting Rhizosphere Microbiome as Alternative Biofertilizer in Boosting Solanum melongena L. Adaptation to Salinity Stress
Source: Plants (Basel). 2022 Feb 28;11(5):659. doi: 10.3390/plants11050659 (PMC8912713; doi:10.3390/plants11050659)
Supplement: Supplementary file 1 [file plants-11-00659-s001.zip › plants-1596842-supplementary.pdf]

Table S1. Effect of 0 (C), selected bioinoculum (I), 200 mM NaCl (S) and their interactions on plant height (PH), root length (RL), shoot length (SL), root/shoot ratio (R/S), root fresh weight (RFW), shoot fresh weight (SFW), total fresh weight (TFW) of *Solanum melongena* L. plant 66 days after sowing. Values are means ± SD based on triplicate independent determinations, and different letters means significant difference as evaluated by Duncan's multiple comparison test ($p < 0.05$).

Treatment	Growth parameters						
	PH (cm)	RL (cm)	SL (cm)	R/S	RFW ($g\ plant^{-1}$)	SFW ($g\ plant^{-1}$)	TFW ($g\ plant^{-1}$)
C	$73.50^{a} \pm 5.20$	$22.50^{a} \pm 3.91$	$51.0^{a} \pm 7.76$	$0.45^{bc} \pm 0.15$	$34.17^{a} \pm 0.76$	$45.18^{a} \pm 13.40$	$79.35^{a} \pm 12.65$
I	$73.83^{a} \pm 13.05$	$22.0^{a} \pm 4.82$	$51.83^{a} \pm 9.70$	$0.43^{c} \pm 0.09$	$13.57^{b} \pm 4.08$	$42.50^{a} \pm 5.96$	$56.07^{b} \pm 1.90$
S	$56.17^{b} \pm 5.58$	$26.0^{a} \pm 2.65$	$30.17^{b} \pm 3.25$	$0.86^{a} \pm 0.06$	$1.41^{d} \pm 0.16$	$7.22^{b} \pm 0.16$	$8.62^{d} \pm 0.30$
S+I	$59.83^{ab} \pm 1.53$	$24.17^{a} \pm 4.25$	$35.67^{b} \pm 3.01$	$0.69^{ab} \pm 0.18$	$8.79^{c} \pm 2.35$	$21.66^{b} \pm 5.15$	$30.46^{c} \pm 7.47$

Table S2. Effect of 0 (C), selected bioinoculum (I), 200 mM NaCl (S) and their interactions on root dry weight (RDW), shoot dry weight (SDW), total dry weight (TDW), root water content (RWC), shoot water content (SWC), total water content (TWC), and leaf area(LA) of *Solanum melongena* L. plant 66 days after sowing. Values are means ± SD based on triplicate independent determinations, and different letters means significant difference as evaluated by Duncan's multiple comparison test ($p < 0.05$).

Treatment	Growth parameters						
	RDW ($g\ plant^{-1}$)	SDW ($g\ plant^{-1}$)	TDW ($g\ plant^{-1}$)	RWC ($g\ plant^{-1}$)	SWC ($g\ plant^{-1}$)	TWC ($g\ plant^{-1}$)	LA (cm^2/plant)
C	$11.35^{a} \pm 0.75$	$7.95^{a} \pm 2.25$	$19.31^{a} \pm 3.0$	$22.81^{a} \pm 1.49$	$37.23^{a} \pm 11.15$	$60.04^{a} \pm 9.66$	$136.5^{a} \pm 25.45$
I	$3.62^{b} \pm 0.76$	$6.88^{ab} \pm 0.74$	$10.50^{b} \pm 0.61$	$9.94^{b} \pm 3.35$	$35.62^{a} \pm 5.40$	$45.56^{b} \pm 2.20$	$122.0^{a} \pm 21.35$
S	$0.42^{c} \pm 0.07$	$1.34^{c} \pm 0.17$	$1.76^{d} \pm 0.23$	$0.99^{c} \pm 0.10$	$5.88^{b} \pm 0.15$	$6.87^{d} \pm 0.17$	$55.78^{b} \pm 9.38$
S+I	$2.47^{b} \pm 0.85$	$4.52^{b} \pm 1.08$	$6.99^{c} \pm 1.93$	$6.32^{b} \pm 1.50$	$17.14^{b} \pm 4.08$	$23.47^{c} \pm 5.56$	$108.6^{a} \pm 32.64$

Table S3: Similarity percentages and accession numbers obtained after comparing the sequence of the tested strain (**B1SRZS**) to the submitted sequences in Gene Bank

Description	Max Score	Total Score	Query Cover	E value	Per. Ident	Accession
Bacillus subtilis subsp. stercoris	1249	1249	52%	0.0	99.71%	MT214144.1
Bacillus subtilis	1249	1249	52%	0.0	99.71%	MT673811.1
Bacillus subtilis	1254	1254	53%	0.0	99.71%	AY973269.1
Bacillus subtilis	1253	1253	53%	0.0	99.56%	MT111030.1
Bacillus siamensis	1256	1256	53%	0.0	99.71%	KX660755.1

Description	Max Score	Total Score	Query Cover	E value	Per. Ident	Accession
Bacillus subtilis	1254	1254	53%	0.0	99.71%	MT111001.1
Bacillus velezensis	1254	1254	53%	0.0	99.71%	MT078629.1
Bacillus amyloliquefaciens	1253	1253	53%	0.0	99.56%	MT081100.1
Bacillus subtilis	1253	1253	53%	0.0	99.56%	MN227486.1
Bacillus tequilensis	1253	1253	53%	0.0	99.56%	MF077125.1
Uncultured bacterium	1253	1253	53%	0.0	99.56%	KP843085.1
Bacillus siamensis	1253	1253	52%	0.0	99.71%	MW474847.1
Bacillus subtilis	1251	1251	52%	0.0	99.71%	MH359177.1
Bacillus sp.	1251	1251	52%	0.0	99.71%	MH769161.1
Bacillus sp.	1251	1251	52%	0.0	99.71%	MH769061.1
Geobacillus sp.	1251	1251	52%	0.0	99.71%	KP792636.1
Bacillus subtilis	1251	1251	52%	0.0	99.71%	MH210872.1
Bacillus sp.	1251	1251	53%	0.0	99.56%	MN826383.1
Bacillus subtilis	1251	1251	52%	0.0	99.71%	MN330078.1
Bacillus amyloliquefaciens	1251	1251	52%	0.0	99.71%	JF899282.1
Bacillus licheniformis	1249	1249	52%	0.0	99.71%	MT525234.1
Bacillus subtilis	1249	1249	52%	0.0	99.71%	MT372156.1
Bacillus velezensis	1249	1249	52%	0.0	99.71%	MT365117.1
Bacillus subtilis subsp. stercoris	1249	1249	52%	0.0	99.71%	MT332715.1
Bacillus velezensis	1249	1249	52%	0.0	99.71%	MT124527.1
Bacillus amyloliquefaciens	1249	1249	52%	0.0	99.71%	MK743994.1
Bacillus sp.	1249	1249	52%	0.0	99.71%	MN826422.1
Bacillus sp.	1249	1249	52%	0.0	99.71%	MN826418.1
Bacillus sp.	1249	1249	52%	0.0	99.71%	MN826404.1
Bacillus subtilis	1245	1245	52%	0.0	99.56%	MW513386.1
Bacillus subtilis	1249	1249	52%	0.0	99.71%	MT898543.1

Table S4: Similarity percentages and accession numbers obtained after comparing the sequence of the tested strain (**B4SRZS**) to the submitted sequences in Gene Bank

Description	Max Score	Total Score	Query Cover	E value	Per. Ident	Accession
Bacillus subtilis	1256	1256	99%	0.0	99.28%	MH359177.1
Bacillus subtilis	1258	1258	99%	0.0	99.28%	AY162133.1

Description	Max Score	Total Score	Query Cover	E value	Per. Ident	Accession
Bacillus subtilis	1256	1256	99%	0.0	99.14%	MT111030.1
Bacillus siamensis	1262	1262	99%	0.0	99.28%	KX660755.1
Bacillus sp.	1260	1260	99%	0.0	99.42%	GU374120.1
Bacillus subtilis	1260	1260	99%	0.0	99.28%	AY973269.1
Bacillus amyloliquefaciens	1258	1258	99%	0.0	99.14%	MT081100.1
Bacillus velezensis	1256	1256	99%	0.0	99.28%	MT103089.1
Bacillus subtilis	1256	1256	99%	0.0	99.14%	MN227486.1
Bacillus subtilis subsp. stercoris	1256	1256	99%	0.0	99.28%	MN704447.1
Bacillus sp.	1256	1256	99%	0.0	99.28%	MK685119.1
Bacillus subtilis	1256	1256	99%	0.0	99.28%	MH210872.1
Bacillus subtilis	1256	1256	98%	0.0	99.42%	MF061811.1
Bacillus subtilis	1256	1256	98%	0.0	99.42%	MF581448.1
Bacillus subtilis	1256	1256	99%	0.0	99.28%	KX467568.1
Bacillus sonorensis	1256	1256	99%	0.0	99.28%	KT986188.1
Uncultured *bacterium*	1256	1256	99%	0.0	99.14%	KP843085.1
Bacillus sp.	1256	1256	99%	0.0	99.28%	KM823924.1
Bacillus amyloliquefaciens	1254	1254	99%	0.0	99.28%	MT613661.1
Bacillus subtilis subsp. subtilis	1254	1254	99%	0.0	99.28%	MT605298.1
Bacillus subtilis subsp. subtilis	1254	1254	99%	0.0	99.28%	MT605295.1
Bacillus subtilis subsp. subtilis	1254	1254	99%	0.0	99.28%	MT605293.1
Bacillus subtilisstraine	1254	1254	99%	0.0	99.28%	MT111083.1
Bacillus subtilis subsp. stercoris	1254	1254	99%	0.0	99.28%	MN704479.1
Bacillus subtilis subsp. stercoris	1254	1254	99%	0.0	99.28%	MN704475.1
Bacillus sp.	1254	1254	99%	0.0	99.28%	MN493914.1
Bacillus sp.	1254	1254	99%	0.0	99.28%	MN493892.1
Bacillus sp.	1254	1254	99%	0.0	99.28%	MN396123.1
Bacillus sp.	1254	1254	99%	0.0	99.28%	MN826347.1
Bacillus sp.	1254	1254	99%	0.0	99.28%	MT950329.1
Bacillus subtilis	1254	1254	99%	0.0	99.28%	MK878625.1
Bacillus subtilis	1253	1253	98%	0.0	99.42%	HG764607.1

Description	Max Score	Total Score	Query Cover	E value	Per. Ident	Accession
Bacillus subtilis	1251	1251	99%	0.0	99.14%	MW513386.1

Table S5: Similarity percentages and accession numbers obtained after comparing the sequence of the tested strain (**B7SRZS**) to the submitted sequences in Gene Bank

Description	Max Score	Total Score	Query Cover	E value	Per. Ident	Accession
Pseudomonas sp.	1258	1258	76%	0.0	99.57%	KR054995.1
Pseudomonas putida	1258	1258	76%	0.0	99.57%	JQ910873.1
Pseudomonas putida	1264	1264	76%	0.0	99.71%	MT271893.1
Pseudomonas sp. s	1264	1264	76%	0.0	99.71%	MT192506.1
Pseudomonas sp.	1264	1264	76%	0.0	99.71%	MT192454.1
Pseudomonas sp.	1264	1264	76%	0.0	99.71%	MT192448.1
Pseudomonas japonica	1264	1264	76%	0.0	99.71%	MH712954.1
Pseudomonas putida	1264	1264	77%	0.0	99.57%	MF952434.1
Pseudomonas sp. s	1264	1264	76%	0.0	99.71%	MH388435.1
Pseudomonas qingdaonensis	1264	1264	76%	0.0	99.71%	MG589917.1
Pseudomonas putida	1264	1264	76%	0.0	99.71%	MF276640.1
Pseudomonas japonica	1264	1264	76%	0.0	99.71%	KX588594.1
Pseudomonas kilonensis	1264	1264	76%	0.0	99.71%	LC005849.1
Pseudomonas sp.	1264	1264	76%	0.0	99.71%	KF896129.1
Pseudomonas sp.	1264	1264	76%	0.0	99.71%	JN886725.1
Pseudomonas sp.	1264	1264	76%	0.0	99.71%	HE979862.1
Pseudomonas japonica	1264	1264	76%	0.0	99.71%	MT658656.1
Uncultured *bacterium*	1264	1264	76%	0.0	99.71%	HQ121069.1
Pseudomonas putida	1264	1264	76%	0.0	99.71%	AB680123.1
Pseudomonas putida	1264	1264	76%	0.0	99.71%	EU439423.1
Pseudomonas putida	1264	1264	76%	0.0	99.71%	AM411059.1
Pseudomonas kilonensis	1260	1260	76%	0.0	99.57%	LC005850.1
Pseudomonas japonica	1260	1260	76%	0.0	99.57%	MT645252.1
Pseudomonas japonica	1260	1260	76%	0.0	99.57%	MT645250.1

Description	Max Score	Total Score	Query Cover	E value	Per. Ident	Accession
Pseudomonas japonica	1260	1260	76%	0.0	99.57%	MT645248.1
Pseudomonas putida	1258	1258	76%	0.0	99.57%	MH379722.1
Uncultured *Pseudomonas sp.*	1258	1258	76%	0.0	99.57%	LN875100.1
Pseudomonas putida	1258	1258	76%	0.0	99.57%	AB681704.1
Pseudomonas sp.	1258	1258	76%	0.0	99.57%	MT672631.1
Uncultured *bacterium*	1258	1258	76%	0.0	99.57%	GU553791.1
Pseudomonas putida	1258	1258	76%	0.0	99.57%	FJ430676.1
Pseudomonas sp.	1256	1256	76%	0.0	99.42%	LC107147.1
Pseudomonas putida	1258	1258	76%	0.0	99.71%	HM641753.1
